# Special Issue “Body Sensors Networks for E-Health Applications”

**DOI:** 10.3390/s20143944

**Published:** 2020-07-16

**Authors:** David Naranjo-Hernández, Javier Reina-Tosina, Laura M. Roa

**Affiliations:** Biomedical Engineering Group, Department of Signal Theory and Communications, University of Seville, 41092 Seville, Spain; jreina@us.es (J.R.-T.); lroa@us.es (L.M.R.)

**Keywords:** BSN, e-Health, on-body antenna, 2.4 GHz, UWB, WNSN, bioimpedance spectroscopy, objective pain assessment, pressure sensors, fall detection

## Abstract

Body Sensor Networks (BSN) have emerged as a particularization of Wireless Sensor Networks (WSN) in the context of body monitoring environments, closely linked to healthcare applications. These networks are made up of smart biomedical sensors that allow the monitoring of physiological parameters and serve as the basis for e-Health applications. This Special Issue collects some of the latest developments in the field of BSN related to new developments in biomedical sensor technologies, the design and experimental characterization of on-body/in-body antennas and new communication protocols for BSN, including some review studies.

## 1. Introduction

The monitoring and analysis of physiological variables through biomedical sensors is fundamental for the diagnosis and monitoring of users and/or patients in the context of e-Health. A biomedical sensor is usually located on the patient to record and analyze physiological signals such as the electrocardiogram, oxygen saturation, blood pressure, body temperature, respiratory rate, heart rate or blood glucose concentration, which can be performed on a 24/7 continuous monitoring scheme (24 h a day, 7 days a week). The biomedical sensors are connected wirelessly to each other or to an external gateway device, forming a body sensors network (BSN). BSNs enable real-time, ubiquitous, pervasive and non-obstructive monitoring of the patient’s health status and the detection of emergency situations. They provide a reduction in the cost of medical care, since monitoring is done outside the clinical setting, and results in an improvement of the quality of diagnosis and medical follow-up, making possible the early diagnosis of a possible disease and the cost-effective management of patients outside the hospital.

These sensors are usually small and lightweight, wearable (placed on the skin or in a garment), but also implantable, to allow non-intrusive monitoring, performed in a seamless way, so that the user can obtain an actual measurement of the physiological variable, without it being affected by the measurement process itself, and avoiding any type of discomfort to the user.

Despite the advances in BSN, there are still many challenges to be addressed, such as the miniaturization of sensor devices for seamless monitoring, usability and scalability, energy efficiency and energy harvesting to provide greater autonomy, the standardization of low-power wireless communication, design and characterization issues related to antennas in a body environment or the integration of implantable devices.

This Special Issue collects some of the latest developments in the field of BSN, covering a wide range of related topics, some of which are listed below:

- Novel and enhanced sensors for physiological measurement
- Innovative health-sensing technologies and applications
- Implantable and minimally invasive devices and nanosensors
- BSN hardware platforms for e-Health applications
- Artifact correction and enhanced monitoring using information fusion
- New processing algorithms and machine learning in BSNs
- Low-power wireless communication technologies for BSNs
- Design and characterization of antennas in BSNs
- Energy efficiency and energy harvesting for body sensor devices
- Big data challenges and Internet of Things in BSNs
- Future challenges of BSNs in e-Health applications

From a total of 14 submissions, 9 articles were finally published after a rigorous peer-review process. A brief introduction to the works published in the Special Issue is made below. The works have been grouped according to their subject in different sections, to provide a better structure and facilitate the follow-up to the reader.

## 2. Articles Related to Sensor Technologies in the Context of BSN and e-Health

Some of the works published in the Special Issue have been related to biomedical sensing technologies. In Reference [1], the design and validation of a new bioimpedance sensor device for the estimation of body composition is presented. Bioimpedance technology has many practical advantages that favor its use in biomedical sensors—non-invasive, repeatable measurements as many times as desired, provide information about the internal physiological processes of the subject under study. The clinical usefulness of bioimpedance for the estimation of the user’s nutritional and hydration status has been demonstrated in numerous studies related to nutrition, chronic diseases, pregnancy and lactation, oncology, monitoring of the elderly, evaluation of athletes, and so forth. The device proposed in Reference [1] addresses some of the challenges of bioimpedance technology related to the precision of the measurements, the use of high-frequency signals (close to MHz) and the energy efficiency. Contrary to the standard measurement technique that uses a single frequency, the proposed device is based on bioimpedance spectroscopy, a more robust and precise technique. Other features of the device are its low cost compared to equivalent precision systems on the market, its bidirectional wireless communication capability, and a semi-automatic self-calibration procedure that favors the adaptability of the system. The work also addresses one of the most complex problems related to bioimpedance measurements: the presence of artifacts that disturb the measurements at low, and, above all, at high frequency. For their solution, the authors propose a modification of the standard Cole model that incorporates two additional dispersions, at low and high frequencies, and a linear phase delay with frequency to take into account the influence of possible parasitic effects. From this model, a computationally efficient algorithm is proposed to identify the model parameters. This algorithm takes advantage of the particular characteristics of the bioimpedance model to obtain better precision and processing speed performance compared to the standard algorithms used for this purpose: nonlinear least-squares methods, optimization of bacteria in search of food, particle swarm or genetic algorithms. The adequacy of the model and algorithm was tested by means of a clinical study in renal patients, where it was highlighted that in the presence of artifacts in bioimpedance measurements, the model and the proposed algorithm provided values more appropriate to the patient’s clinical situation than the bioimpedance device used as the reference.

Fakhrulddin et al. [2] describes another biomedical sensing system in the BSN environment. This system is applied to the detection of falls in the elderly, which have a high and increasing incidence rate. This work proposes an advanced first aid system for elderly people in a context of an outdoor environment. The system is based on a BSN with sensors to measure accelerations, used in the detection of falls, and to monitor the heart rate, as a consequence of the high rate of occurrence of atrial fibrillation in the falls, associated with dizziness, fainting and the reduction of the amount of oxygen in the brain in elderly people in situations of weakness or instability. The system described in Reference [2] proposes a hybrid detection algorithm that uses both accelerations and heart rate measurement. The sensor prototype, located on the upper part of the person’s arm, includes the two bio-sensors, a microcontroller where the hybrid algorithm is run, a GPS (Global Positioning System) module for the person’s geolocation and a GSM module (Global System for Mobile Communications) to send a notification message to a call emergency center (CEC) once a fall and abnormal heart rate are detected. To address the context of application to the monitoring of the person anywhere, including outdoors, a GSM communications module has been used. The proposal is complemented by a first aid provision system based on an unmanned aerial vehicle (UAV) that carries a package of first aid supplies from the CEC to the person who has suffered a fall. For the route planning, an intelligent autopilot program that uses the subject’s geolocation as a reference point is used. The first aid packages are adapted according to the state of the subject who has suffered the fall. The experimental results showed good performance of the system in the measurement of heart rate, the detection of falls, the accuracy of the GPS location and the improvements in saving time in the emergency assistance to the user.

The system described in Reference [3] proposes a sensor technology based on sensors integrated in insoles to be placed on shoes for the development of a pedometer adapted for use in elderly people. Pedometers are commonly used for the evaluation of energy expenditure and physical activity in rehabilitation activities, patient monitoring and training. However, the precision of these systems, normally based on accelerometers, has been limited in studies conducted in elderly people or patients with pathologies related to brain damage. To address this problem, the authors of Reference [3] propose an insole with five force-sensitive resistances to measure the foot pressure. Based on the information provided by the sensors, a new method to accounting for the number of steps is proposed. Common methods typically employ averaging or summation of pressure signals, which can result in dual signal peaks resulting from delayed heel and forefoot support, and therefore overestimation of the step count. Instead, the proposed method is based on the cumulative sum of the pressure data obtained in the foot support phase in the gait cycle, which generates a single pressure peak. The results confirmed the adequacy of the system and the method to be used in elderly people and considering different walking speeds.

## 3. Articles Related to the Design and Characterization of Antennas In BSN

BSN communications typically focus their application on the 2.4 GHz frequency band, reserved internationally for industrial, scientific and medical (ISM) use. Therefore, a critical element in BSNs are the antennas, especially in biomedical applications where size restrictions and energy efficiency are essential. Despite the fact that in these types of applications the human body has a capital influence on the performance of the antenna, its effects are not normally taken into account in the design and experimental characterization of antennas for communications on the human body (on-body) or inside the human body (in-body). The study carried out in Reference [4] describes a procedure for the experimental characterization of antennas for battery-powered devices inside an anechoic chamber and under the influence of the human body. In this situation, the standard passive measurement procedure in an anechoic chamber, using a network analyzer and coaxial cables to feed the antenna, may not be the most suitable option. The procedure proposed in Reference [4] addresses the solution of this problem in an comprehensive way—(1) incorporating all the hardware elements that may affect the performance of the antenna compared to the standard approach that analyzes antenna in an isolated way; (2) integrating the transceiver device powered by battery versus the passive characterization via coaxial cable, thus removing the mismatches caused by impedance mismatch; (3) using a spectrum analyzer instead of a network analyzer to solve problems arising from the physical level of the communications protocol. The method also describes the procedure for the analysis of the effects of the human body, and proposes the use of a body phantom for the automatic evaluation of the radiation characteristics inside an anechoic chamber, without the limitations derived from the difficulty of movement and orientation of the human body. The procedure was applied to the design and characterization of four different antennas. The simulation results using an electromagnetic analysis software confirmed the adequacy of including all the elements that make up the hardware in the simulation model to provide results closer to actual measurements. However, electromagnetic simulation tools do not allow these elements to be easily incorporated. The measurements obtained with the proposed procedure provided a realistic evaluation of the experimental results. The results obtained with the phantom were equivalent to the measurements on the human body confirming its adequacy. Moreover, a configuration in which the antenna is slightly separated from the body provides better performance with respect to another in which it is directly attached to the body.

The Special Issue has included another work related to the design and characterization of antennas for biomedical devices [5]. In this case, the antenna was designed for the intrabody communications of an endoscopic capsule applied to video examinations of the digestive tract. In contrast to many of the systems described in the literature, which place the antennas on the outer surface of the capsules, the antenna presented in this work is arranged inside the capsule. In this way, the losses derived from the biocompatible material of capsule covering layer are controlled and the manufacturing process is simplified. The architecture of the capsule is made up of stackable modules comprising the battery, the camera and the transceiver. The antenna design is ring-shaped, allowing it to be integrated together with the endoscopic camera in the same module. The radiating element of the antenna is a dipole tuned to cover the 2.4 GHz band, with a meandered structure to reduce size. The experimental evaluation was carried out by introducing the endoscopic capsule in a deionized water container, with a conductivity and a dielectric constant similar to muscle tissue at the frequencies of interest. The dipole structure of the antenna facilitated the correction of the deviations observed in the tuning of the antenna when it was introduced into the phantom. An analysis of the radiation characteristics of the antenna inside an anechoic chamber showed an omni-directional behavior, close to that of the isotropic antenna, which is ideal for the intrabody use of the endoscopic capsule. Furthermore, compared to other antennas in the literature, the antenna bandwidth was greater.

## 4. Articles Related to Communication Protocols in the Field of BSN

Communication protocols are another aspect of interest in BSNs. In this sense, the study presented in Reference [6] proposes an energy efficient routing protocol for intrabody wireless networks of biomedical sensors based on nanotechnology. Thanks to new materials such as carbon nanotubes and graphene, new sensorization opportunities are opening up in the field of biomedicine, taking advantage of their size, sensitivity and biocompatibility. These materials operate at frequencies close to terahertz, but this is an underused spectrum that can have a great impact on the future development of medical technologies, since it allows a smaller size of the antennas, a greater bandwidth, a lower sensitivity to dispersion and propagation effects, and greater safety in biological mediums. Wireless NanoSensor Networks (WNSN) address the communication problems of nano-sensors and nano-devices. In this type of network, the measurement is performed by a large group of nano-sensors distributed in the area of interest. Furthermore, the nodes have very limited energy and computational resources. These aspects condition communications, which must be carried out cooperatively and be managed using routing protocols of low complexity and high energy efficiency. The protocol described in Reference [6] proposes a new clustering method to solve the problems derived from the high number and short communication range of the nano-nodes in a WNSN inside the human body. This protocol continuously updates the main node of the cluster between the nodes with the highest residual energy, balancing the power consumption of the network. The clustering of the nodes is done in layers so that a data packet can be sent to the main node of the cluster in one hop. The number of nodes in the cluster can be reduced in order to decrease the overall energy consumption of the network. Finally, the main nodes of the cluster communicate with a control node through multi-hop routing. The simulation results highlight the advantages of the proposed protocol over other recent protocols, guaranteeing energy efficiency and the success rate in the information transmission.

The study carried out in Reference [7] focuses on the external access of the BSNs, placing special interest in the analysis of the influence of the human body on this type of wireless communication. Recent research papers are investigating the improvements provided by the dynamic adaptation to conditions of line-of-sight (LOS) or non-line-of-sight (NLOS). In this way it is possible to minimize the packet error rate by dynamically adapting the transmission bit rate depending on the visibility conditions caused by the changing nature of the communication channel due to the presence of the human body. The protocol proposed in Reference [7] is based in Ultra-Wideband (UWB) technology because of its robustness in multipath conditions versus narrowband options, and uses a low complexity Deep Neural Network (DNN) (only two hidden layers, each with 50 nodes) that can be run in real time in a standard general-purpose microcontroller. The experimental results confirmed the benefits of DNN in classifying LOS and NLOS conditions in different experiments carried out with volunteers. Based on these results, it could be concluded that body structure and body mass index (BMI) are factors that affect the effectiveness of the classification algorithm, being lower in subjects with higher BMI. Furthermore, the suitability of self-learning technologies for their application in radio communication systems is confirmed.

## 5. Review Articles in the Context of BSN

Finally, some works have carried out reviews of sensor technologies compatible with BSN. In this sense, the authors of Reference [8] carry out a detailed review of the different technologies that have been proposed for the objective evaluation of chronic pain, which is prevalent in multiple pathologies. The chronic pain condition is directly related to the patient’s well-being, and also to the psychological state (anxiety, depression, etc.). The standard evaluation methods of chronic pain are subjective and self-reported evaluations performed by the patients, the medical staff or the caregivers. However, due to their subjective nature, these reports are sometimes imprecise and may lead to insufficient or inappropriate therapy. Objective pain assessment is challenging for researchers, and different approaches for pain measurement based on biomedical sensors have been proposed, although to date there is no universal method for the objective pain assessment. The referenced work [8] reviews and comparatively analyzes the different methods described in the literature focusing on chronic non-cancer pain. One of the variables employed in the studies is the heart rate variability (HRV), since pain influences the balance of the autonomic nervous system (ANS), detectable through the analysis of HRV. Commonly, people with chronic pain have a decreased HRV compared to subjects without pain. In addition, the analysis of the spectrum of the HRV also reveals a clear reduction of the high-frequency components. The review work also examines studies that have analyzed the relationships between physical activity and pain, since patients who have chronic musculoskeletal pain are usually less active. It is common that the anxiety derived from pain sensation causes the subjects to acquire a habit of self-protection and avoidance of pain-related movement. Another parameter of interest is skin conductance, since pain can produce an increase in sweat production triggered by the ANS, and consequently, the conductance. Electromyogram (EMG) signals are also related to the presence of pain due to the increase in muscle tension. Other techniques are based on computer vision and image processing, since the facial features associated with pain are highly identifying. However, these methods are complex and expensive, and unsuitable for use during the patient’s daily life. The work also reviews other sensor technologies, such as the measurement of respiratory rate since pain can trigger an irregular breathing pattern, blood pressure that can increase in stages of pain, and body temperature that can decrease as a result of vasoconstriction produced by pain, among others.

In the review work carried out in Reference [9], different methods for the measurement and evaluation of the pillars that contribute to brain health are analyzed. This work is justified as a consequence of the increase in life expectancy, associated with a higher prevalence and incidence of psychiatric, neurological and cognitive deterioration pathologies. Although age is a risk factor, it is not the trigger factor and interventions based on healthy lifestyle habits can contribute to the preservation of brain integrity, mental well-being, and cognitive function in advanced ages. The factors that most influence the health of the brain are physical exercise, nutrition and adequate sleep, although other factors such as the maintenance of cognitive activity, a vital plan, general health and social interactions are also of importance. Monitoring these pillars is key in identifying factors that may influence the brain health of a particular subject, and in designing personalized interventions to maintain mental well-being and prevent the cognitive decline. Biomedical sensors integrated into BSN can be used for this purpose, since they are small, portable and allow wireless communication with smartphones for the collection of information. In Reference [9] a systematic review of the literature that analyzes the relevant parameters for mental health monitoring is carried out, analyzing which parameters can help to modify and improve people’s habits, the results obtained with the application of these technologies, and the target populations of the different studies. The study results show that there are pillars such as sleep, socialization or cognitive activity that have rarely been studied from the aspect of brain health. Other aspects analyzed in the study were the methods used in the monitoring of the subjects, the technology that supports the monitoring and the type of intervention carried out, including machine learning methods such as neural networks or machine learning for the elaboration of personalized and adapted interventions. The results of the work indicate that more exhaustive studies are needed to confirm the value of physiological monitoring and personalized interventions in the area of brain health.

## 6. Conclusions

Through this special issue the published papers show that BSN is a broad multidisciplinary area with a clear growth potential. The maturity of technology is increasing the scope of applications. BSN is becoming a pusher for the advancement of the e-Health paradigm and contributing to the shift of healthcare from a reactive to a preventive medicine practice. 

## Abbreviations

The following abbreviations are been used:

ANS	Autonomic Nervous System
BMI	Body Mass Index
BSN	Body Sensor Network
CEC	Call Emergency Center
EMG	Electromiogram
GPS	Global Positioning System
GSM	Global System for Mobile Communications
HRV	Heart Rate Variability
ISM	Industrial, Scientific and Medical
LOS	Line-of-Sight
NLOS	Non-Line-of-Sight
UAV	Unmanned Aerial Vehicle
UWB	Ultra-Wideband
WNSN	Wireless NanoSensor Network
WSN	Wireless Sensor Networks
